# Evaluation of the Effectiveness of Brazilian Community Restaurants for the Dimension of Low-Income People Access to Food

**DOI:** 10.3390/nu13082671

**Published:** 2021-07-31

**Authors:** Mateus Santana Sousa, Camila Silveira Silva Teixeira, Jamacy Costa Souza, Priscila Ribas de Farias Costa, Renata Puppin Zandonadi, Raquel Braz Assunção Botelho, Heesup Han, António Raposo, Antonio Ariza-Montes, Luis Araya-Castillo, Rita de Cássia Coelho de Almeida Akutsu

**Affiliations:** 1 Escola de Nutrição, Universidade Federal da Bahia, Salvador 40110-909, Brazil; mattheus_sousa@outlook.com (M.S.S.); jamacy@ufba.br (J.C.S.); prfarias@ufba.br (P.R.d.F.C.); rita.akutsu@gmail.com (R.d.C.C.d.A.A.); 2 Instituto de Saúde Coletiva, Universidade Federal da Bahia, Salvador 40110-040, Brazil; camilasilveira.nutri@gmail.com; 3 Department of Nutrition, Faculty of Health Sciences, University of Brasilia, Brasilia 70910-900, Brazil; renatapz@unb.br (R.P.Z.); raquelbotelho@unb.br (R.B.A.B.); 4 College of Hospitality and Tourism Management, Sejong University, 98 Gunja-dong, Gwanjin-gu, Seoul 143-747, Korea; 5 CBIOS (Research Center for Biosciences and Health Technologies), Universidade Lusófona de Humanidades e Tecnologias, Campo Grande 376, 1749-024 Lisboa, Portugal; 6 Social Matters Research Group, Universidad Loyola Andalucía, C/Escritor Castilla Aguayo, 4, 14004 Córdoba, Spain; ariza@uloyola.es; 7 Faculty of Business Administration, Universidad Autónoma de Chile, Santiago 7500912, Chile; 8 Facultad de Economía y Negocios, Universidad Andrés Bello, Santiago de Chile 7591538, Chile; luis.araya@unab.cl

**Keywords:** low income, community restaurants, effectiveness, food access, food assistance program

## Abstract

This study aimed to evaluate the effectiveness of community restaurants (CRs), managed by the Government of the State of Bahia/Brazil, for the dimension of access to food. The study used secondary data obtained from the public opinion survey Profile of users of community restaurants in Salvador. The nutritional information was accessed through the analysis of CRs’ menus. Adequate effectiveness of access to food was considered when the CR served meals to 50% to 70% of the users considered the target audience (individuals served by the two CRs located in the city of Salvador/Bahia/Brazil). The participants (*n* = 1464; 778 as low-income individuals) were adult CR users from Salvador/Brazil. Most of the respondents were male, 40 to 54 years old, not white, had up to 9 years of formal education, without a partner, and living in the municipality of Salvador. The evaluated CRs are effective in serving 53.1% of the target population in their total service capacity. Meal provision only reached an estimated 0.7% of the socially vulnerable community in the district. The average energy value of the meal served by the CR units was 853.05 kcal/meal, with a mean energy density composition classified as average (1.15 kcal/g). The effectiveness of the evaluated community restaurants showed that these instruments were minimally effective in promoting access to food for the low-income population within their total daily service capacity, and the current quantity of these facilities was insufficient. However, these instruments stand out in the fundamental role of promoting the daily distribution of meals to the Brazilian population with the highest social vulnerability levels.

## 1. Introduction

In Brazil, a considerable expansion of social security and development programs has been observed in the last two decades [1]. The Zero Hunger Program, created in 2003, was guided by a broad effort to fight hunger and misery in the country. The program was constituted by a set of actions and strategies, with the primary objective of guaranteeing opportunities for equality and social inclusion with equity for the population with greater vulnerability [2]. Moreover, some emergency and structural policies were highlighted, which made it possible to implement programs for continuous access to food such as school meals, distribution of basic food baskets, food banks, and community restaurants [2,3]. The Community Restaurants Program (CR) was created in 2003 as a public food assistance policy, guided by the Food and Nutrition Security Guidelines and financed by the Ministry of Social Development and Fight against Hunger in Brazil [2,4,5]. The CR proposes to offer healthy meals at affordable prices (USD 0.15 to USD 1.51) [6], managed by the public sector. Access to CR is universal, but those exposed to a greater level of social vulnerability and poverty situations are priority users. Therefore, they are located in all of the five Brazilian regions, primarily where populations with higher risks of food and nutritional insecurity are concentrated, also contributing to the local food system [2,7].

Among the Brazilian regions, the northeast presents the country’s highest poverty rates. It is marked by socioeconomic problems, reflected in vast social inequalities and development difficulties amplified by the severe climatic conditions of long periods of drought [8]. Therefore, this region presents 27% (*n* = 41) of the Brazilian CRs, distributed in 9 of the 26 states in Brazil (Alagoas, Bahia, Ceará, Maranhão, Paraíba, Piauí, Pernambuco, Rio Grande do Norte, and Sergipe). In this sense, it is essential to evaluate this policy in serving the target population. Social groups’ health status is directly related to the context in which they live and their position in the social pyramid. Understanding these contexts can contribute to Food and Nutrition Security’s theme, especially for these most vulnerable groups [9]. 

The evaluation of programs and public policies has high relevance and is directly related to these strategies’ effectiveness, efficiency, and accountability. Effectiveness, particularly, concerns the relationship between the results and the objective by measuring the impact or the degree of achievement of the proposed objectives [10,11,12]. Assessing effectiveness is an indicator of the effects of the program in the place where it is inserted. The primary focus is to detect changes in the access conditions of a target group or community due to a program in which the changes occurred in the desired direction [13].

In recent decades studies have sought to identify and characterize different management models, target audience, the number of meals served, type of menus offered, the origin of food, sources of funds, costs, and profile of users of Brazilian CR [5,14,15,16,17,18,19,20,21,22,23]. The results of these assessments can support decision making for rational budget allocation and program reorganization to achieve planning objectives [12]. However, none of them evaluated the effectiveness of the Brazilian community restaurants for the dimension of low-income individuals’ access to food. Therefore, we aimed to investigate the effectiveness of CRs managed by the Government of the State of Bahia/Brazil for the dimension of low-income people’s access to food.

## 2. Materials and Methods

### 2.1. Study Design

This is a cross-sectional, descriptive study using secondary data obtained from the public opinion survey entitled “Profile of users of community restaurants in Salvador.” The survey was conducted by the Secretary of Justice, Human Rights, and Social Development of the Government of the State of Bahia, Brazil (*SJDHDS*) [24]. All the information used in this study was derived from secondary data provided by the public survey and from the dietitians of the two CRs.

### 2.2. Participants

The target population considered for the study included individuals served by the only two CRs from Salvador/Bahia/Brazil. The state government manages the two units (Liberdade and Comércio), and the service covers the entire local population without public selection. These CRs have easy access by the population and are located in neighborhoods with the worst social indicators [25,26]. In these CRs, 4945 meals are daily offered at lunchtime, on a 5-day-a-week basis (Monday to Friday). 

For this study, we defined “target audience” as individuals with self-reported monthly per capita income estimated at up to ½ minimum wage (considering one minimum wage equivalent to USD 141.97 per month) [27], recognized as low-income people and in a situation of social vulnerability [14,22]. Participants were individuals of both genders, aged ≥ 18 years old.

The sample universe of 4945 individuals was considered for population estimation, represented by the total number of daily meals distributed in both CRs. The sample calculation was performed through the census survey of gender and age quotas in the respective CR. A 95% confidence level and an acceptable error of 3% were considered to obtain a more robust sample. The sample was composed of a minimum of 863 users. Therefore, a minimum of 863 respondents of the public opinion survey was necessary to represent the population that eats at the CR.

### 2.3. Data Collection

The Brazilian Institute of Public Opinion (IBOPE) designed a questionnaire based on a survey to identify and characterize the different modalities of CR in Brazilian cities with more than 100,000 inhabitants [14]. General characteristics of the household, income, education, and head of the household were evaluated in this questionnaire. Additionally, social support, work situation, lifestyle, perception of health status and satisfaction, and service use were included. IBOPE pretested the questionnaire to check for potential problems, language adjustments, as well as an estimated average interview time.

In this study, we inserted all the data collected by IBOPE and analyzed the attendance percentage of customers considered a priority by the CR program. For the second part of data collection, assessment of nutritional aspects, researchers requested CRs’ dietitians to send their monthly menus with the average amount of food in grams of each served dish (rice, beans, main protein dish, garnish, and dessert) as well as the technical preparation files (TPFs) of all the dishes for nutrients’ calculation. Since CR opens from Monday to Friday, researchers received TPFs and portions’ sizes from 20 menus that complete a monthly lunch menu.

### 2.4. Study Variables

For this study, researchers used the following characteristics of the target audience available from the secondary data of the public opinion survey: age in years; gender (male, female, transgender); self-declared race/skin color (white, not white); education (no schooling; 5 years of study; 9 years of study; 12 years of study; ≥13 years of study); marital status (no partner; married/stable relationship, divorced), residency in Salvador (yes or no), and beneficiary of social programs (yes or no).

The program’s effectiveness was assessed as the primary outcome, estimated by the coverage proportion of the “target audience”, defined within the scope of the CR Program. We considered adequate effectiveness of access to food when the CRs, within their capability, served meals to 50% to 70% of the users considered the “target audience”, as suggested by Hartz and Silva (Table 1) [28]. In this sense, CR was considered effective for the dimension of access to food, as they presented a coverage percentage of the target audience equal to or greater than 50%. In particular, in this study, the effectivity reflects the direct and indirect impacts of the services offered by the public action, that is, it deals with the effective changes carried out in that reality that is the object of the program (individuals in a situation of food and nutritional insecurity). 

Additionally, a calculation was performed to estimate the CR program’s necessary coverage in serving low-income people, considering only the city where the survey was conducted. We considered a total of 715,000 people classified as socially vulnerable based on population estimates from the Brazilian Institute of Geography and Statistics (IGBE) for the municipality of Salvador/Bahia/Brazil [29] for the calculation. Therefore, we divided the total number of users by the entire low-income population in the city.

To assess nutritional aspects, we calculated the total energy value (Kcal), carbohydrates, proteins, lipids, and sodium of each lunch served in each CR, and the average of all the meals offered in a month. Dietbox Professional 2014^®^ was used for calculations. The energy density of the dishes was calculated and classified according to the nutritional criteria of the American Center for Disease Control and Prevention (CDC), namely, high energy density (4 to 9 kcal/g), average energy density (1.5 to 4 kcal/g), low energy density (0.7 to 1.5 kcal/g), and very low energy density (0 to 0.6 kcal/g) [23,30].

### 2.5. Data Analysis

The descriptive analysis was performed by absolute and relative frequencies and their respective 95% confidence intervals (95% CI). Some variables were recategorized to present the results. The Stata 15.0 program (Stata Corporation, College Station, USA) was used for the analysis.

### 2.6. Ethical Aspects

This project was approved by the Ethics Committee of the School of Nutrition of the Federal University of Bahia (No. 3534763), following the National Ethics and Research Commission [31]. No personally identifiable information was included in the data set used for analysis.

## 3. Results

The study sample information extracted from the public opinion survey was composed of 1464 individuals—778 low-income individuals and 686 as other CR users. Table 2 shows the socioeconomic profile of the participants. Most participants declared themselves as not white (blacks, browns, indigenous, and yellow) (92.2%), from 40 and 54 years old (26.7%), up to 9 years of formal education (51.5%), and without a partner (56.1%). Most of the interviewees declared to live in Salvador (95.9%), and 18.9% were beneficiaries of other social programs.

The proportion of CR’s general effectiveness in serving low-income individuals was estimated at 53.1% (95% CI: 50.6–55.7) considered average. For the other users, the estimation was at 46.9%. The current coverage of CR to serve the socially vulnerable population in Salvador was estimated at 0.7%. 

With the TPF and the mean portions of all the dishes served in the CR, researchers calculated meals’ macronutrients, energy value, total grams of the portioned plate, and ED. The average TEV of the lunch meals served by the researched CR was 853.05 kcal/meal, with energy density (ED) classified as low (1.15 kcal/g) (Table 3). There are no statistical differences among ED for the monthly meals (*p* = 0.49). The mean carbohydrate level of the meals was 109.19 ± 24.84 g (51.0% of TEV), protein level was 40.37 ± 11.90 g (18.9% of TEV), and lipids were 28.62 ± 13.46 g (30.1% of TEV). The daily mean sodium of the lunch meal was 1235.32 ± 1069.48 mg. Sodium was calculated by the information from the TPF based on the sodium content of the ingredients. Extra salt added by users while eating at the CR was not considered since researchers used secondary data for this study.

## 4. Discussion

The evaluated CRs in the city of Salvador, which represent the CR Program of the Government of the State of Bahia, showed effectiveness considered at the lower limit of adequacy for the dimension of access to food for the target audience. The current coverage of serving the low-income population was less than 1%, which was expected since the CRs are located only in the state’s capital. The lunch meal offered by the CRs exceeded the energy intake per meal recommended by national and international feeding programs [32,33]. However, it is very close to the 800 kcal recommended by the Brazilian Worker Feeding Program, showing medium energy density. Macronutrient distribution follows the IOM recommendations [34], but sodium offer exceeds the recommendation for lunch intake (40% of the total daily intake) [22]. Even though mean ED was classified as medium, the percentage of lipids in the user’s plate is within the recommendations for the adult population.

Access to healthy and adequate food in its nutritional aspect is a fundamental and inherent right to human dignity. Thus, it is up to the state to elaborate its Food and Nutritional Security Policy, establishing permanent food policies and programs, considering national sovereignty and the Federal Constitution, structuring it through health promotion principles and strategies, with the formulation of permanent public policies [35]. According to the United Nations (ONU) new report, about 821 million people worldwide have little or no access to food, and they are in a hunger situation. In Brazil, a significant portion of the population (6.8 million) does not have sufficient access to food to maintain their health daily, reflecting a critical situation of food and nutritional insecurity [36].

In the Brazilian scenario, diverse situations of greater vulnerability are observed for food insecurity, especially in the north and northeast regions, which, in 2017, had the lowest average income in the country [5]. In the State of Bahia, in that same year, the percentage of people below the poverty line (44.8%) was above the national average (26.5%). In addition to the significant percentage of people below the poverty line in Bahia, 3.3% of households did not have any per capita income [29]. All these lines of evidence demonstrate a vast demand for social policies to serve the population. They indicate that a significant portion of the population lives without minimum access to food security along CR policy lines. 

The ideal coverage estimation of the CRs showed that only 0.7% of the low-income population of the city of Salvador would be served, which demonstrates a great weakness of this policy in the state since these units represent the totality of CRs managed by the state government for the city of Salvador. These two CR units were created in 2001 and 2002, and they were incorporated into the national program in 2003. In 2003, there were around 111 CRs in Brazil, but there was an increase of 37% in the CR numbers by 2014 [6,14]. They contributed to this program’s increase in covered areas and improved health indicators guaranteeing food and nutritional security (FNS) [20,37]. From that period on, the federal government financed 46% of FNS programs in the country, and the State Government of Bahia built no new units [24].

We estimate that to meet the total demand, taking into account only the capital, which differentially has about 715,000 people in low-income situations, it would be necessary to implant approximately 140 CRs with an average capacity of 5000 meals/day. Additionally, according to the literature, it would be important to partner with other sectors, such as integrating other public food policies, income transfer programs, and access to jobs aimed at this low-income population [7,35]. 

Based on the FNS guidelines, in addition to guaranteeing access, food must be offered with decent quality and continuously, through the provision of at least two daily meals, which must consider aspects of a cultural and survival nature, with nutrients capable of keeping the body functioning [38]. The intake must be carried out in quantities necessary to meet the nutritional needs since some nutrients are not cumulative and must be consumed daily to meet the physiological needs [38]. In this sense, based on the Brazilian Food Guide, the national food policy provides that food should be offered through intersectoral actions whose main objective is to improve the population’s food and nutrition standards, especially those of low income, to contribute to health promotion [38,39].

Comparing the total energy value served in the meal of the evaluated CRs (853.05 Kcal/meal), an adequate energy intake is observed, covering around the caloric value recommended by the Worker Food Program, for example [32]. This value is very close to the average daily intake recommended by the Brazilian Food Guide [39]. 

In general terms, FAO estimates a caloric value of 2000 to 2500 Kcal/day for an adult man and 1500 to 2000 Kcal/day for women to maintain a healthy life [33]. For Silva [35], food intake should not be less than 1800 Kcal/day. It would be considered an insufficient intake, characterizing a situation of hunger, as it would not be enough to satisfy the minimum daily energy needs.

The average energy density of the menu (lunch) observed in this study was 1.15 kcal/g, lower than the average observed for CRs in Brazil (1.34 kcal/g) [22]. The average observed in this study is even lower than the values observed in other studies that varied from 1.25 kcal/g [40] to 1.98 kcal/g [41]. High values of energy density are related to the poor quality of the diet, which can contribute to the increase of excess weight [41]. Considering such evidence, national and international organizations recommend avoiding high-energy-density foods or meals concentrated in few meals [14,42]. Our study observed that the menu had a low energy density, which is necessary for low-income people who consume little or have no food. Additionally, they often have no means to acquire food other than what CRs offer at lunch in other menus [43]. Thus, the fractional supply of other meals during the day can substantially impact access to food for this population. Faced with this scenario, we found that planning the number of meals/day in these surveyed units is insufficient, as they serve only one meal per day (lunch) and do not work during weekends and holidays. This fact was also verified in a nationwide study, which highlighted lunch as the main meal consumed by users of CRs distributed among the five major regions of Brazil [22]. 

The monthly mean of sodium was 1235.32 ± 1069.48 mg, lower than the results found by Carrijo et al. [22] in a study evaluating Brazilian CRs. However, it is important to mention that the sodium content is almost half of the sodium recommendation for the entire day [44], impacting chronic diseases development. It is essential to highlight that the sodium content might be underestimated since we did not consider the sachets of salt, which are available for users. CRs often use pork sausage, pork offal, hot dog sausage, and dried meat, which are cheap but have high sodium and fat content, influencing the results. 

The users’ sociodemographic profile was similar to the profiles of users assessed in previous studies with CRs distributed throughout Brazil. They were predominantly male, aged between 31 and 59 years old, non-white, and most were not beneficiaries of the *Bolsa Família* program. Regarding education, users attended high school or technical courses, differing only in married/stable relationships [5,16,19,20,22,45,46,47]. The sociodemographic and economic characteristics are directly linked to the family’s health condition, considering access to food, goods, and essential services [48,49]. Studies show that factors such as being female, low educated, with insufficient per capita household income, and the federative unit’s regional location, especially in the north and northeast regions, can hinder access to food and favor the occurrence of food insecurity [43,50,51,52].

Comparing the Brazilians’ CR with international programs, such as The Meals Program in the US, which consists of equipment that mixes operation and management (similar to food banks, community kitchens, and CR in Brazil), they are primarily located in large urban centers, and their target audience is homeless people [53,54]. Nutritional problems are also found in the US program, as there is no standard of control concerning the meal served. There is also a high prevalence of nutritional imbalances [53]. This fact may be correlated, as restaurants usually receive and serve an abundance of leftover cakes, pizzas, and desserts donated by local restaurants. This fact can raise the caloric value of meals, as well as increase the levels of carbohydrates and lipids, and consequently lead to lower levels of fiber, calcium, potassium, and vitamins [55].

This study of community restaurants in Brazil aimed to bring contributions targeting the administrators of these programs, adding scientific, academic, social, and cooperation knowledge between researchers. This study presents some limitations as the use of secondary data, which can limit the inclusion of important variables such as the nutritional evaluation of the population, the situation of food insecurity, and its main difficulties regarding access to food to assess the effectiveness of the CR. Still, the possibility of inserting other indicators and measures in the evaluation model for a more complete and specific measurement of CR’s effectiveness is highlighted. It is reinforced that the evaluation matrix elaborated is subject to change and future improvements. Another limitation is the impossibility to discuss the obtained results with solutions used in other countries because none of them present a program with objectives and guidelines similar to those observed in the CR Brazilian program.

## 5. Conclusions

The proportion of effectiveness observed among the evaluated community restaurants showed that these instruments were minimally effective in promoting access to food for the population considered a target audience—low-income people. Additionally, it is necessary to increase the supply of food access, by expanding the CR number. The areas in which the units are located in the State of Bahia, specifically in the Municipality of Salvador, have vast social inequalities and consequently access to food. The greater reach of the target audience and the CR installation in the State’s interior should make this policy more effective as an instrument to promote access to food throughout the territory. Nutritionally, CRs are offering an adequate energy value meal, with an average ED and adequate distribution of macronutrients. The sodium offer is above the recommendations. In addition, it is recommended to map food-insecure people to assess the coverage of the policy. This mapping should be used as an active search for people with greater vulnerability. Thus, it would be possible to discuss the improvement of the current policy and the inclusion/adoption of a food assistance model based on successful programs in other countries. It is noteworthy that the CR has a fundamental role in accessing food and achieving the Human Right to Adequate Food and Food and Nutritional Security for populations with higher levels of social vulnerability. Therefore, they must be understood as instruments of great importance within the National Policy on Food and Nutritional Security and must support and integrate other social policies.

## Figures and Tables

**Table 1:** Criteria for evaluating the effectiveness of health programs and systems.

Coverage Percentage	Effectiveness Classification	
<50%	Low	Not very effective
50 to 70%	Adequate	Effective
>70%	High	Excellent effectiveness

Source: Hartz and Silva [28].

**Table 2:** Socioeconomic profile of users of popular restaurants (*n* = 1464), Salvador/Bahia/Brazil.

Variable	*n*	*P* (%)	CI 95%
Age range (years)			
18–24	95	6.5	5.3–7.8
25–39	306	20.9	18.8–23.0
40–54	391	26.7	24.5–29.0
55–64	306	20.9	18.8–23.0
≥65	366	25.0	22.8–27.2
Gender			
Female	373	25.5	23.3–27.7
Male	1087	74.2	71.9–76.4
Transgender	4	0.3	0.1–0.7
Skin color			
White	112	7.8	6.4–9.2
Not withe	1329	92.2	90.7–93.5
Not informed	23		
Education level (years of study)			
Without study	70	4.8	3.8–6.0
5 years of study	299	20.5	18.5–22.6
9 years of study	382	26.2	24.0-28.5
12 years of study	615	42.1	39.6–44.7
≥13 years of study	94	6.4	5.2–7.8
Not informed	4		
Marital status			
Without a partener	819	56.1	53.5–58.6
With a partner	315	21.6	19.5–23.7
Divorced	326	22.3	20.2–24.5
Not informed	4		
Live in Salvador			
Yes	1404	95.9	94.7–96.8
No	60	4.1	3.1–5.2
A beneficiary of other social programs besides the community restaurant			
Yes	276	18.9	16.9–20.9
No	1184	81.1	79.0–83.0
Not informed	4		

*n* (%): absolute and relative frequency; *P* (%): prevalence; CI 95%: confidence interval of 95%.

**Table 3:** Mean energy value (kcal), meal plate weight (grams), and energy density (ED—kcal/grams) of the monthly menus from the community restaurants, Bahia/Brazil.

Days of the Week	Mean Kcal ± SD	Mean Grams ± SD	Mean ED ± SD (kcal/g)
Monday	878.75 ± 291.36	765 ± 105.44	1.21 ± 0.61
Tuesday	833.50 ± 102.19	821.25 ± 30.92	1.02 ± 0.12
Wednesday	894 ± 195.37	687.75 ± 96.82	1.30 ± 0.20
Thursday	898 ± 212.17	742.50 ± 83.12	1.20 ± 0.20
Friday	761 ± 161.38	767.50 ± 89.95	1.01 ± 0.31
Weekly mean	853.05 ± 168.30	756.80 ± 48.24	1.15 ± 0.13

## Data Availability

The study did not report any data.
